# Supplementary material for: Elastocaloric, barocaloric and magnetocaloric effects in spin crossover polymer composite films
Source: Nat Commun. 2024 Jul 22;15:6171. doi: 10.1038/s41467-024-50373-2 (PMC11263356; doi:10.1038/s41467-024-50373-2)
Supplement: Supplementary file 1 — Supplementary Information [file 41467_2024_50373_MOESM1_ESM.pdf]

Supplementary information to: Elastocaloric, Barocaloric and Magnetocaloric effects in Spin Crossover Polymer Composite Films

Klara Lünser[1], Eyüp Kavak[2,3], Kübra Gürpinar[3,4], Baris Emre[2], Orhan Atakol[4], Enric Stern-Taulats[1], Marcel Porta[5], Antoni Planes[1], Pol Lloveras[6], Josep-Lluís Tamarit[6], and Lluís Mañosa[1,*]

[1]Departament de Física de la Matèria Condensada, Facultat de Física. Universitat de Barcelona, Martí i Franquès, 1, Barcelona, 08028,Catalonia.
[2]Department of Engineering Physics, Faculty of Engineering. Ankara University, Ankara, 06100, Türkiye.
[3]Graduate School of Natural and Applied Sciences. Ankara University, Ankara 06100, Türkiye.
[4]Department of Chemistry, Faculty of Science. Ankara University, Ankara, 06100, Türkiye.
[5]Departament de Física Quàntica i Astrofísica, Facultat de Física. Universitat de Barcelona, Martí i Franquès, 1, Barcelona, 08028,Catalonia.
[6]Grup de Caracterització de Materials. Departament de Física, EEBE and Barcelona Research Center in Multiscale Science and Engineering. Universitat Politècnica de Catalunya, Eduard Maristany, 10-14, Barcelona, 08019, Catalonia.
[*]Corresponding author: lluis.manosa@fmc.ub.edu

1 Chemical Formula of the Spin Crossover Complex

The complex used in this work was reported in [1] . It is a Fe(II) molecular spin crossover compound, $[FeL_2][BF_4]_2$ [L = 2,6-di(pyrazol-1-yl)pyridine] in which Fe(II) ions are connected to 2,6-di(pyrazol-1-yl)pyridine ligands. Two BF_4^- counter anions are present in each formula unit.

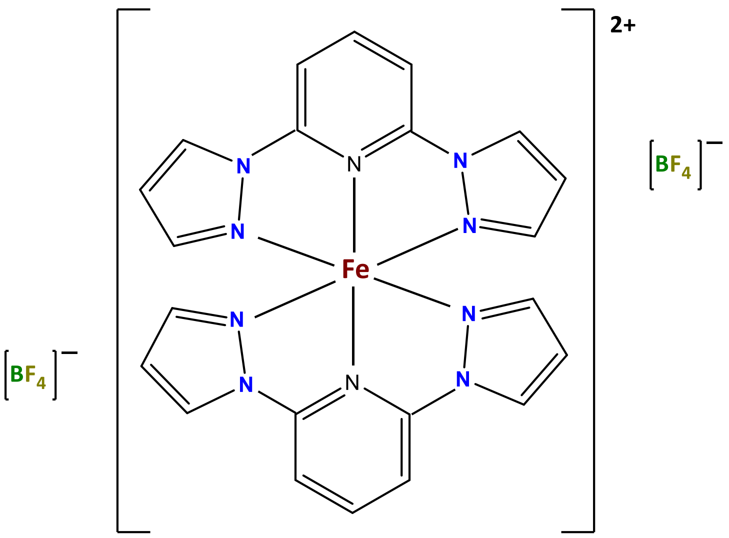

Figure S1: Chemical formula of the $[Fe(L)_2](BF_4)_2$, [L=2,6di(pyrazol-1-yl)pyridine] Spin crossover complex (**1**)

2 Infrared spectra

(a)

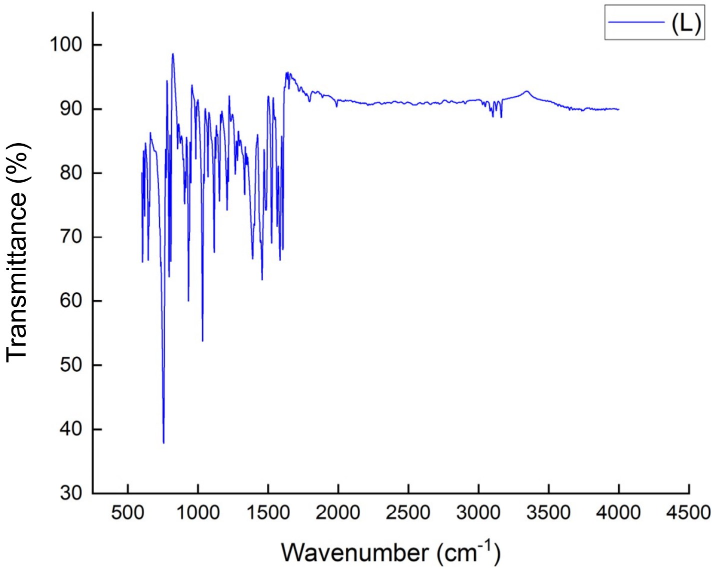

(b)

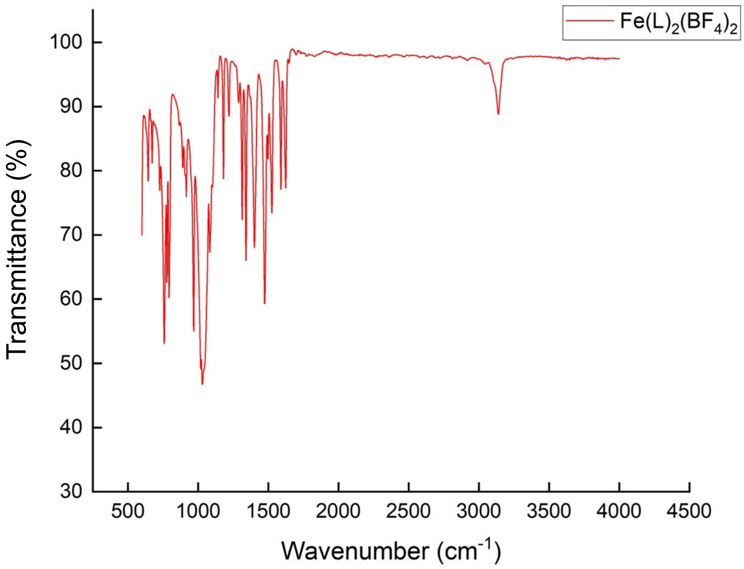

Figure S2: Room temperature Infrared spectra. (a) the Ligand (L=(bis-2,6(pyrazol-1-yl)pyridine)) in cm^{-1}: 3110-3165 ν(C-H)$_{Ar}$; 1603 ν(C=N)$_{ring}$; 1583 ν(C=C)$_{ring}$; 1479-1458 ν(C-N); 763 δ(C-H). (b) for $Fe(L)_2(BF_4)_2$, in cm^{-1}: 3112-3146 ν(C-H)$_{Ar}$; 1624 ν(C=N)$_{ring}$; 1589 ν(C=C)$_{ring}$; 1525-1473 ν(C-N); 1083-1031 ν(B-F); 758 δ(C-H)

3 Nuclear Magnetic Resonance

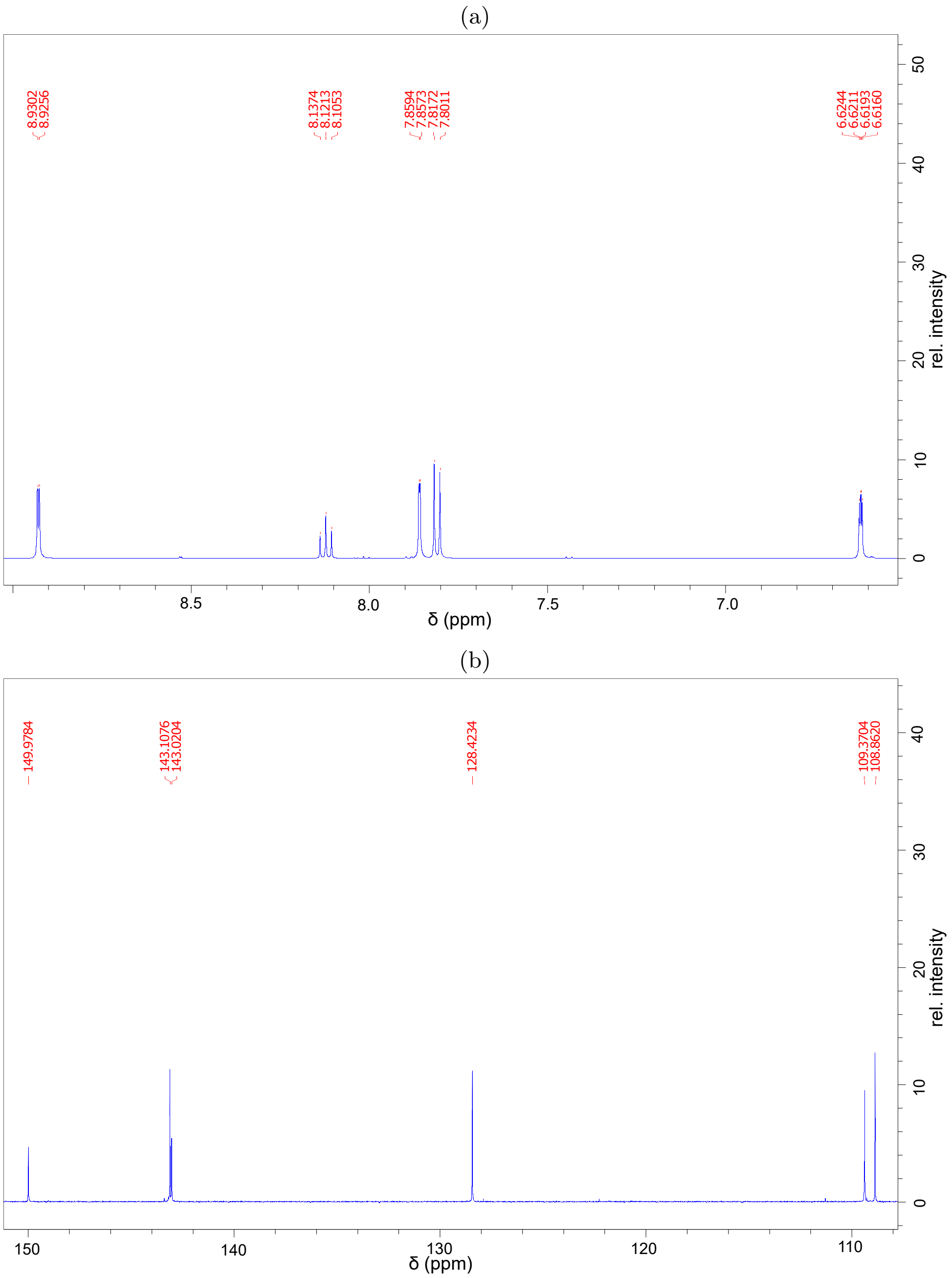

Figure S3: ^{1}H (a) and ^{13}C (b) NMR spectra of the Ligand (L=(bis-2,6(pyrazol-1-yl)pyridine)). Results are as expected: in ^{1}H NMR spectrum of the ligand, five different signals (3 doublets, two triplets) at 8.93, 8.14, 7.86, 7.82 and 6.62 ppm for five different hydrogen atoms are observed; and in ^{13}C NMR spectrum, six different signals in the aromatic area at 149.98, 143.11, 143.02, 128.42, 109.37 and 108.86 ppm are observed for six different carbon atoms.

4 Characterization of pure PVC

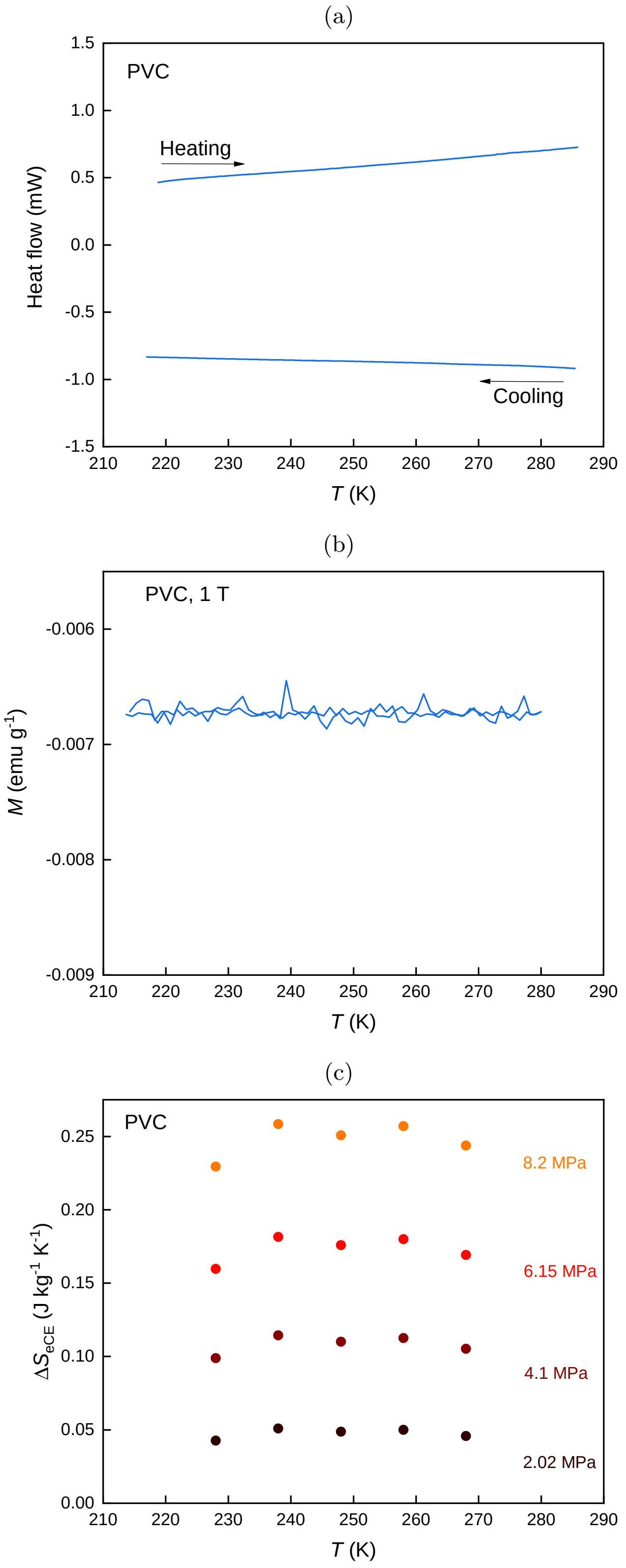

Figure S4: Characteristics of pure PVC film. (a) differential scanning calorimetry, (b) isofield magnetization measured at 1 T and (c) stress-induced elastocaloric isothermal entropy change for selected values of applied uniaxial tensile stress. In all measurements, PVC shows no transformation.

5 Reproducibility of the SCO transition in 1/PVC

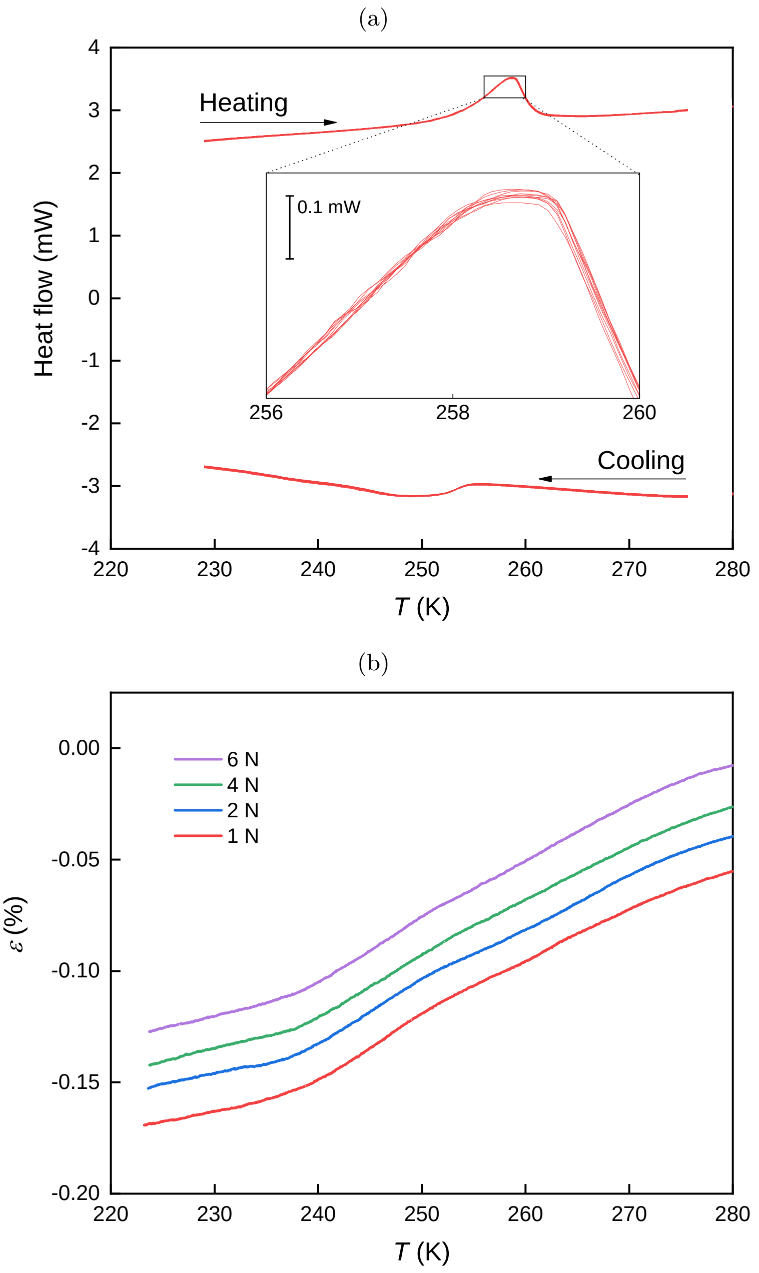

Figure S5: (a) Differential scanning calorimetry measurement of **1**/PVC for 10 subsequent cycles of heating (upper curves) and cooling (lower curves). There is no difference in transformation due to cycling. The inset shows a magnified view were the different curves (for heating runs) are distinguishable. (b) Strain as a function of temperature for **1**/PVC at selected constant forces measured on the same sample. Both measurements (a) and (b) prove the reproducibility of the SCO transition in **1**/PVC.

6 Structural characterization of 1 and 1/PVC

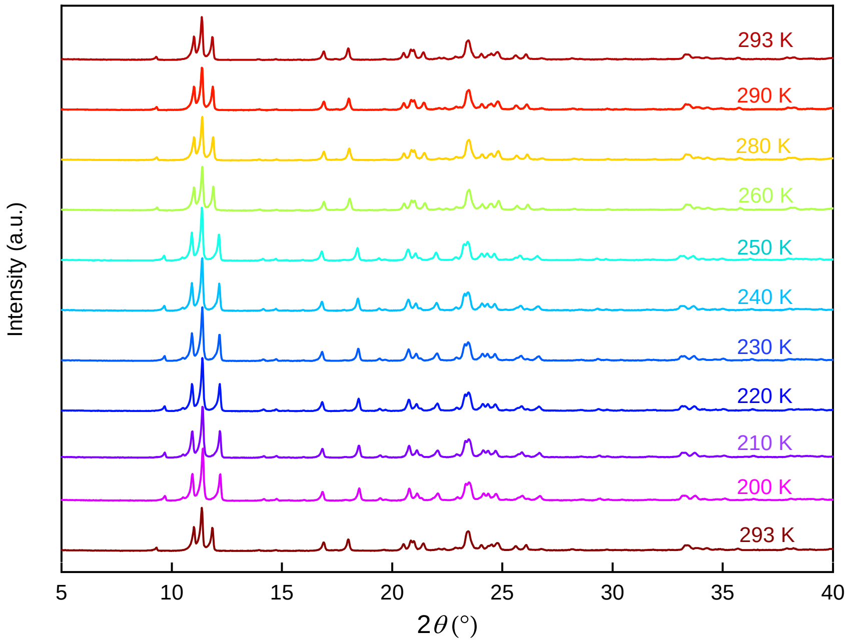

Figure S6: Temperature-dependent x-ray diffraction measurement of **1** powder. After conducting one room temperature measurement (brown), the sample was cooled to 200 K then heated at a rate of 2 K min^{-1}. Isothermal measurements were taken every 10 K. The transformation is visible as a shift in peak position between 250 K and 260 K.

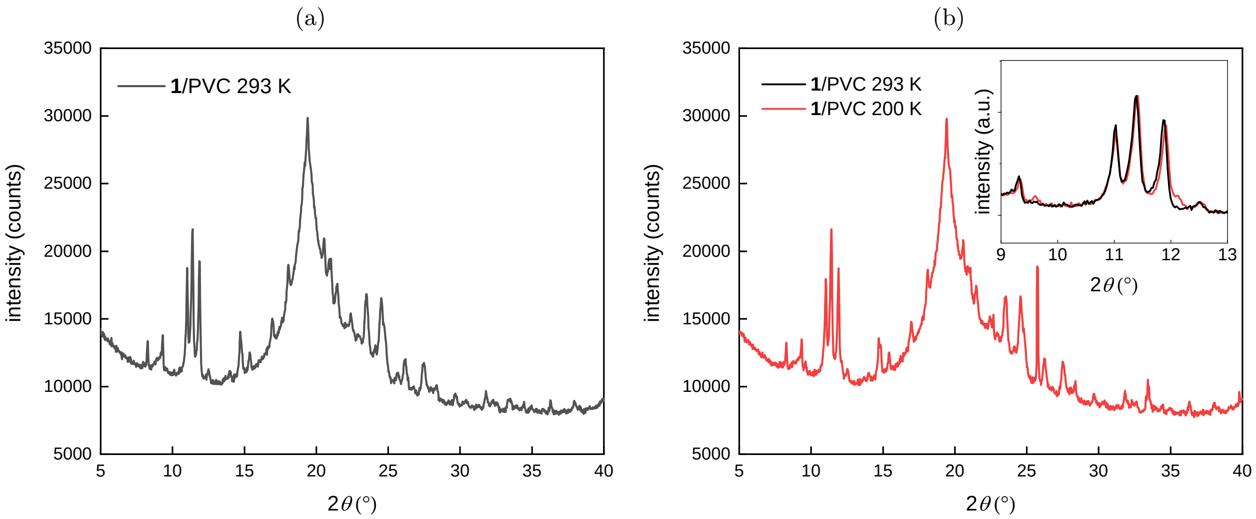

Figure S7: X-ray diffraction (XRD) measurements of **1**/PVC. The measurements at room temperature (a, black curve) and at 200 K (b, red curve) show diffraction peaks of **1** on top of a PVC background. As only a small fraction of **1** transforms during cooling, both XRD measurements are very similar. Small peaks next to the main peaks (see inset in b) belong to the transforming fraction.

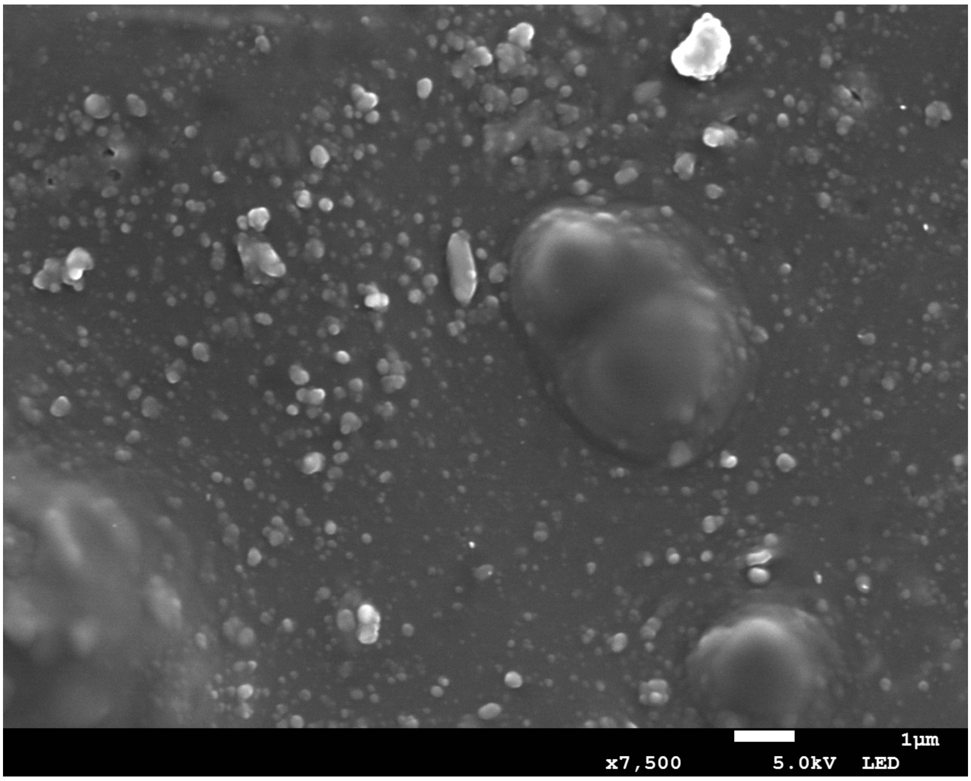

Figure S8: Scanning Electron Microscopy (SEM) image of **1**/PVC showing small SCO particles dispersed in the polymer matrix. The image was taken at an acceleration voltage of 5 kV using secondary electrons.

7 Magnetic measurements in 1 and 1/PVC

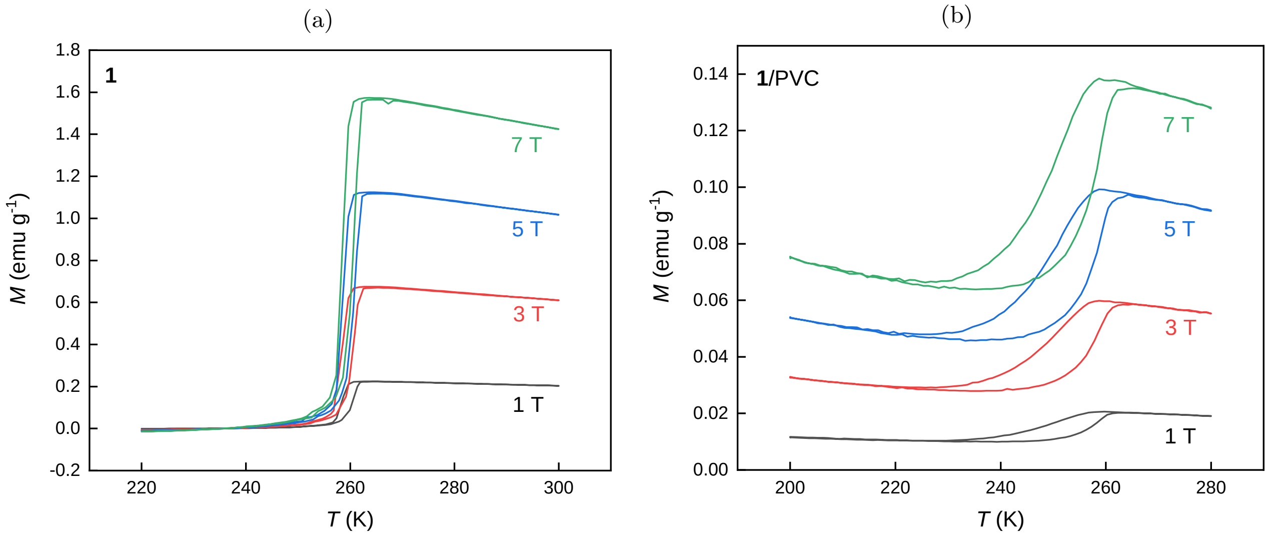

Figure S9: Isofield magnetization measurements at external magnetic fields of 1 T, 3 T, 5 T and 7 T for (a) **1** and (b) **1**/PVC.

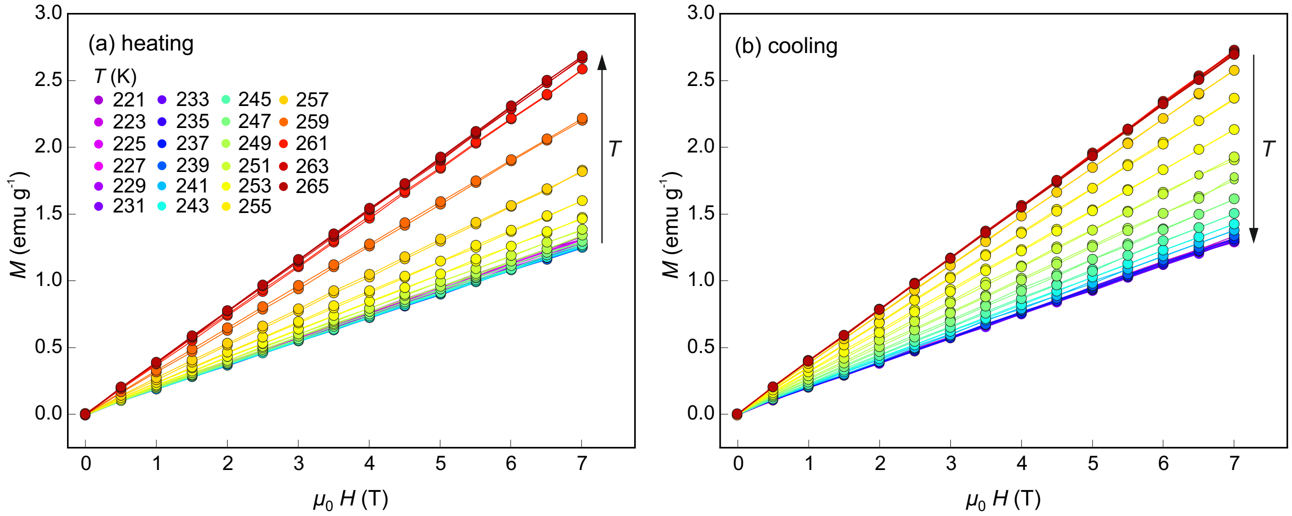

Figure S10: Isothermal magnetization measurements as a function of magnetic field for **1** for selected (a) increasing and (b) decreasing values of temperature. The same colour code applies for the temperature values to the two panels. Lines are guides to the eye.

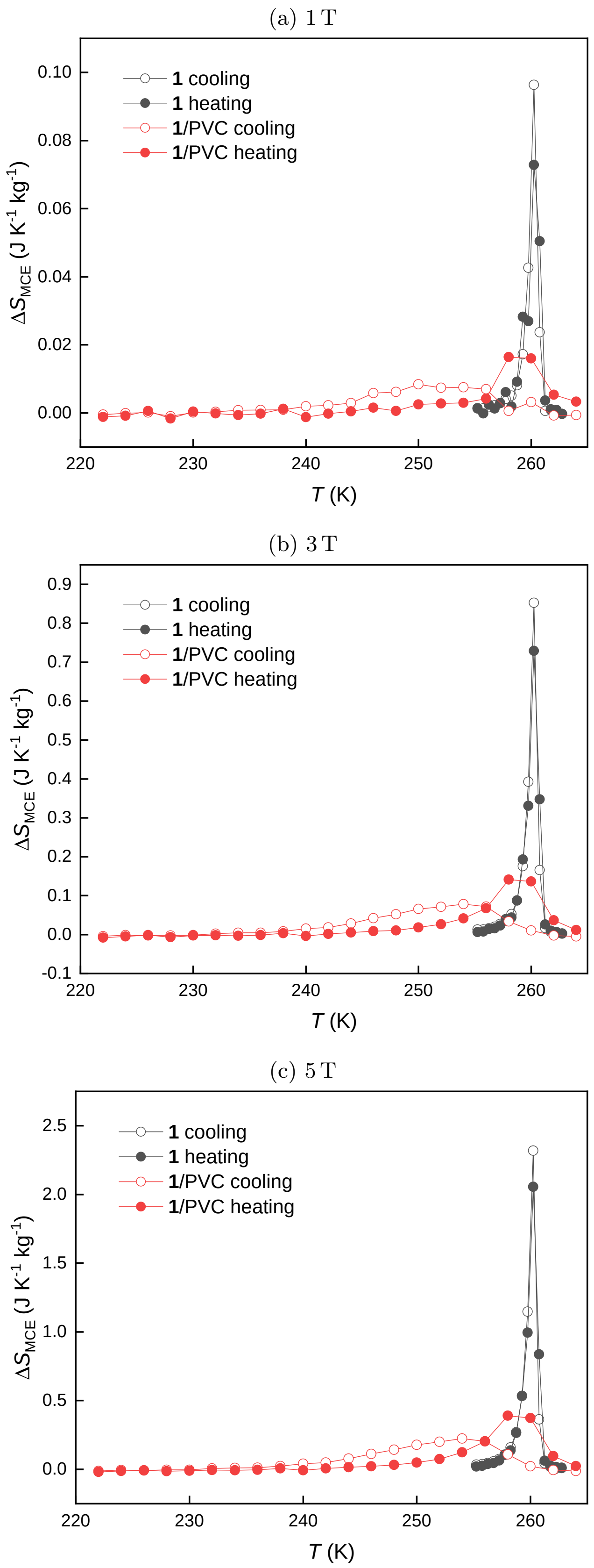

Figure S11: Magnetocaloric isothermal entropy change corresponding to the application of a (a) 1 T, (b) 3 T and (c) 5 T magnetic field for **1** (black symbols and lines) and **1**/PVC (red symbols and lines). Open symbols correspond to decreasing temperatures, while solid symbols correspond to increasing temperatures. Lines are guides to the eye.

8 High pressure differential thermal analysis in 1/PVC

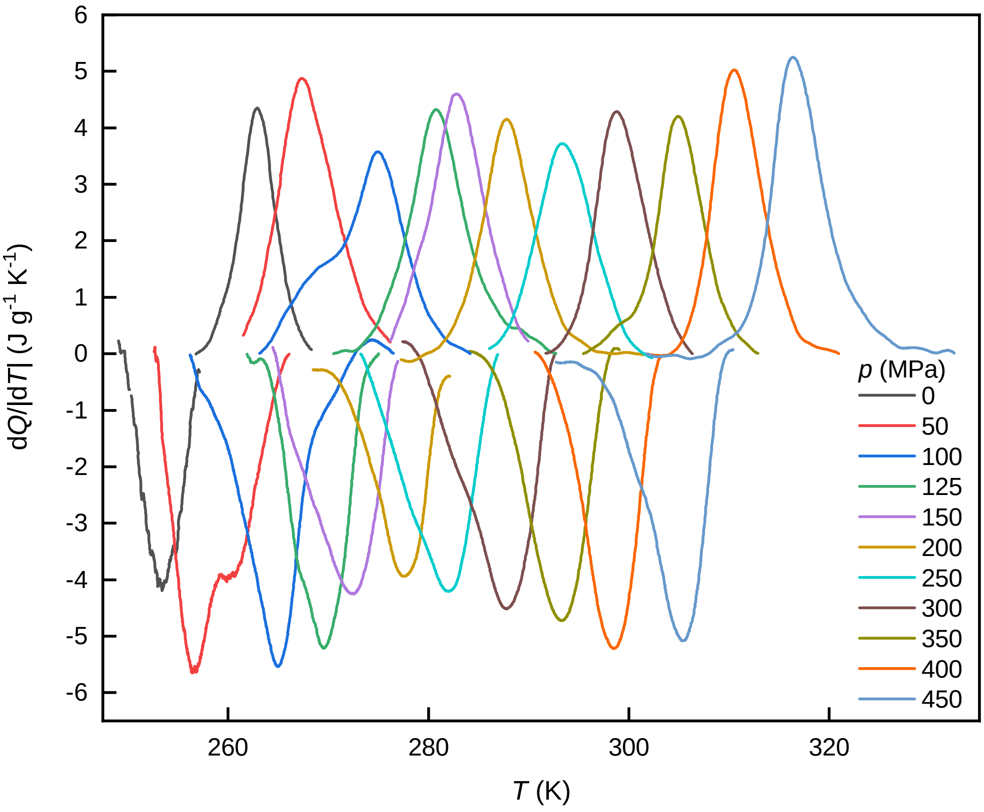

Figure S12: Temperature-dependent isobaric heat flow dQ/|dT| for **1**/PVC, at selected values of hydrostatic pressure, after baseline subtraction. The upper curves correspond to the endothermal LS to HS transition on heating, and the lower curves to the exothermal HS to LS transition on cooling.

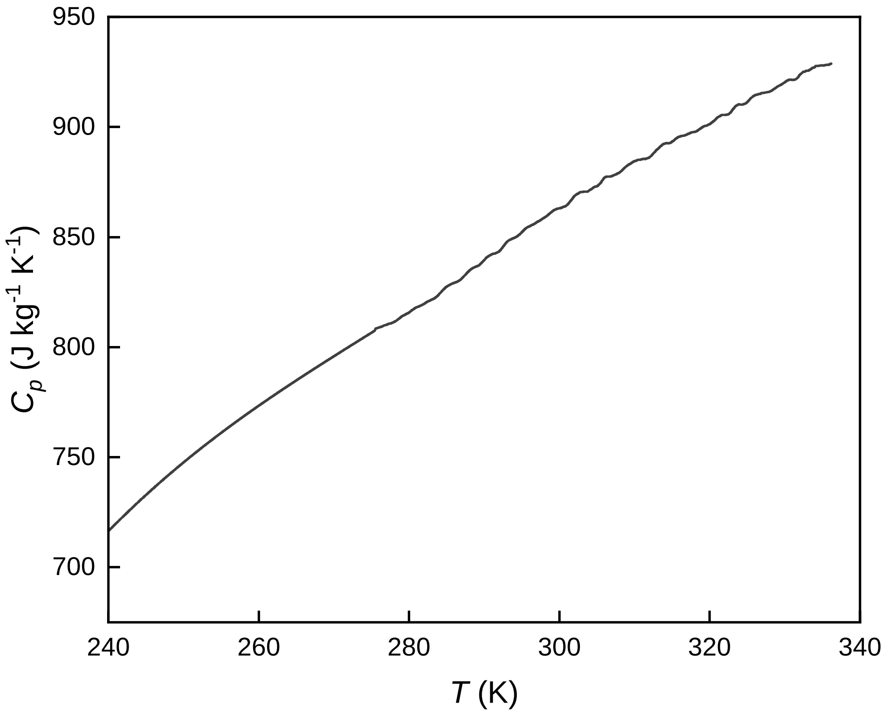

Figure S13: Specific heat of **1** measured with differential scanning calorimetry in modulated mode, with ±0.50 K every 60 s and a heating rate of 1 K min^{-1}. The peak arising from the transition latent heat is excluded.

8.1 Entropy curves

The combination of specific heat data with the pressure-dependent thermal curves enables the determination of the entropy, referenced to a value at a given temperature (T_0) and atmospheric pressure, as:

$$S'(T,p) = \begin{cases} \displaystyle\int_{T_0}^{T} \frac{C_p^{LS}}{T} dT & ; \quad T \leq T_1 \\ \displaystyle S'(T_1,p) + \int_{T_1}^{T} \frac{1}{T}\left(C_p + \frac{dQ}{dT}\right) dT & ; \quad T_1 < T \leq T_2 \\ \displaystyle S'(T_2,p) + \int_{T_2}^{T} \frac{C_p^{HS}}{T} dT & ; \quad T_2 < T \end{cases} \tag{1}$$

where T_1 and T_2 are the start and end temperatures of the SCO transition. C_p^{LS} and C_p^{HS} are specific heats of the LS and HS states, respectively, and $C_p = xC_p^{LS} + (1-x)C_p^{HS}$ where x is the fraction of the sample in the LS state. Equation (1) is computed by assuming pressure-independent specific heats, which is a good approximation, taking into account the relatively narrow temperature interval over which it is computed. Nevertheless, the contribution to the entropy ($\Delta S_+(T_0,p)$) arising from the thermal expansion of each phase cannot be neglected for compressible organic and metal-organic materials. Such a contribution can be computed as:

$$\Delta S_+(T_0,p) = S(T_0,p) - S(T_0,p_{atm}) = \int_0^{T_0} \frac{C_p(T,p) - C_p(T_0,p_{atm})}{T} dT \simeq -\left(\frac{\partial V}{\partial T}\right)_p \Delta p \tag{2}$$

with $\Delta p = (p - p_{atm}) \simeq p$, and where $\left(\frac{\partial V}{\partial T}\right)_p$ is evaluated at $p_{atm} = 1$ atm, and it is assumed to be pressure-independent.

The entropy curves as a function of temperature for selected values of applied pressure are finally computed as: $S(T,p) = S'(T,p) + \Delta S_+(T_0,p)$

9 Model

We use a two-dimensional Ginzburg-Landau model to study an inclusion exhibiting a volumetric phase transition embedded into an elastic matrix. The free energy density is written as an expansion in terms of the so-called

symmetry-adapted strains, which are linear combinations of components of the Lagrangian strain tensor, ε_{ij}. These are the volumetric, $e_1 = (\varepsilon_{xx} + \varepsilon_{yy})/\sqrt{2}$, deviatoric, $e_2 = (\varepsilon_{xx} - \varepsilon_{yy})/\sqrt{2}$ and shear, $e_3 = \varepsilon_{xy}$, strains.

The order parameter of the phase transformation is the volumetric strain, e_1. Thus, within the inclusion the free energy density is expanded in terms of this symmetry-adapted strain up to fourth order, which is the lowest order required to model a first-order phase transition. We also include the quadratic terms on the non order parameter strain components, e_2 and e_3, which are essential to properly model the long range anisotropic elastic interactions. Finally, a gradient term for each symmetry-adapted strain is also included. For simplicity, other gradient terms of the same order that symmetry allows are neglected. Thus, the free energy density within the inclusion is written as

$$\begin{aligned} f_{\text{inc}} = \; & \frac{1}{2}A(T-T_c)e_1^2 + \frac{1}{3}\zeta e_1^3 + \frac{1}{4}\gamma e_1^4 + \frac{1}{2}A_2 e_2^2 + \frac{1}{2}A_3 e_3^2 \\ & + \frac{1}{2}\kappa_1|\nabla e_1|^2 + \frac{1}{2}\kappa_2|\nabla e_2|^2 + \frac{1}{2}\kappa_3|\nabla e_3|^2, \end{aligned} \tag{3}$$

where T is the temperature and T_c is the stability limit of the high temperature phase. A, ζ and γ are related to second and higher order elastic constants of the inclusion, and κ_n represent a contribution to the energy cost of creating variations in the symmetry-adapted strains.

The free energy density of the matrix, which is purely elastic with no phase transition, is simply written as

$$f_{\text{mat}} = \frac{1}{2}A_1 e_1^2 + \frac{1}{2}A_2 e_2^2 + \frac{1}{2}A_3 e_3^2 + \frac{1}{2}\kappa_1|\nabla e_1|^2 + \frac{1}{2}\kappa_2|\nabla e_2|^2 + \frac{1}{2}\kappa_3|\nabla e_3|^2. \tag{4}$$

The parameter A_1 is the bulk modulus $A_1 = C_{11} + C_{12}$. The parameters A_2 and A_3 are related to the second-order elastic constants C' and C_{44}: $A_2 = C_{11} - C_{12} = 2C'$ and $A_3 = 4C_{44}$. For simplicity we simulate an isotropic system and thus we use the relationship $A_3 = 2A_2$. The symmetry-adapted strains are computed from the Lagrangian strain tensor which, in turn, is obtained from the displacement field, $\mathbf{u}(\mathbf{r})$, which is the variable of the model.

The total free energy of the system thus reads,

$$F = F_{\text{inc}} + F_{\text{mat}}, \tag{5}$$

with

$$\begin{aligned} F_{\text{inc}} &= \int_{\Omega} f_{\text{inc}} d\mathbf{r}, \\ F_{\text{mat}} &= \int_{\overline{\Omega}} f_{\text{mat}} d\mathbf{r}, \end{aligned} \tag{6}$$

where Ω is the volume containing the inclusion and $\overline{\Omega}$ is the volume of the system excluding the inclusion.

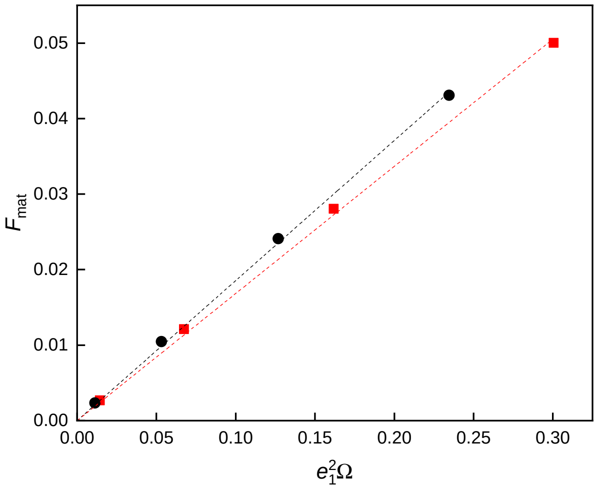

Figure S14: Free energy of the elastic matrix upon transformation of a single isolated inclusion vs $e_1^2\Omega$, where e_1 is the transformation strain and Ω is the volume of the inclusion. Circles correspond to circular inclusions and squares correspond to square inclusions. Dashed lines are linear fits to the numerical data.

For a given shape and size of the inclusion, the total free energy is minimized at $T = 0.8\,T_c$, below the phase transition, and the resulting strain fields are analyzed. For both circular and square inclusions it is obtained

that the strain within the inclusion is almost homogeneous. Thus, the free energy of the inclusion can be approximated to,

$$F_{\rm inc} \approx \left[\frac{1}{2}A(T-T_c)e_1^2 + \frac{1}{3}\zeta e_1^3 + \frac{1}{4}\gamma e_1^4\right]\Omega, \tag{7}$$

as, due to strain compatibility, the non order parameters are negligibly small if the gradient of the order parameter is small.

In Fig. S14 we plot the free energy of the matrix vs $e_1^2\Omega$, where e_1 is the average value of the volumetric strain within the inclusion. We obtain a linear relationship for both circular and square inclusions, although the proportionality constant is different in these two cases. A slight deviation from linearity is attributed to the finite size of the matrix in the simulations.

Thus, the free energy of the matrix can be approximated as,

$$F_{\rm mat} \approx Be_1^2\Omega, \tag{8}$$

where B is a constant that depends on the bulk modulus of the matrix and on the shape of the inclusion. This energy cost leads to a decrease in the transition temperature of the inclusion, in agreement with experimental findings.

The parameters of the model T_c, A, and κ_1 are fixed to unity which defines reduced units of energy, length and temperature. In these reduced units the parameters of the model used to study the free energy of the matrix are $\zeta = 20$, $\gamma = 5000$, $A_1 = 0.05$, $A_2 = 0.1$, $A_3 = 0.2$ and $\kappa_2 = \kappa_3 = 1$. The simulations have been carried out in a system of size $L \times L = 1000 \times 1000$ reduced units of length discretized onto a 512×512 mesh, and the radius of the circular inclusions considered was $R = 20$, 39, 59 and 78 reduced units of length.

References

[1]. J.M. Holland et al., Chem. Commun. 2001, 577.
